# Humanismo en la práctica de médicos cooperantes cubanos en Brasil: narrativas de equipos de atención básica

**DOI:** 10.26633/RPSP.2017.130

**Published:** 2017-12-26

**Authors:** Yamila Comes, Ximena Pamela Díaz-Bermúdez, Lucélia Luiz Pereira, Felipe Proenço de Oliveira, José Emilio Caballero González, Helena Eri Shimizu, Leonor Maria Pacheco Santos

**Affiliations:** 1 Universidad de Brasilia Universidad de Brasilia Brasil Universidad de Brasilia, Brasil.; 2 Universidad Federal de Paraíba Universidad Federal de Paraíba João Pessoa Brasil Universidad Federal de Paraíba, João Pessoa, Brasil.; 3 Organización Panamericana de la Salud Organización Panamericana de la Salud Brasilia Brasil Organización Panamericana de la Salud, Brasilia, Brasil.

**Keywords:** Atención primaria de salud, humanismo, educación médica, cooperación internacional, Cuba, Brasil, Primary health care, humanism, education, medical, international cooperation, Cuba, Brazil, Atenção primária à saúde, humanismo, educação médica, cooperação internacional, Cuba, Brasil

## Abstract

**Objetivo.:**

*Mostrar las narrativas de miembros brasileños de los equipos de salud de la familia acerca del humanismo percibido en la práctica de los cooperantes cubanos del programa* Mais Médicos.

**Métodos.:**

*Estudio de caso descriptivo de corte transversal. Se aplicó una entrevista semiestructurada a miembros brasileños de los equipos de salud de la familia que trabajaran desde el inicio del programa con médicos cubanos en municipios seleccionados inscritos en el programa* Mais Médicos*, con 20% o más de su población en extrema pobreza, y menos de cinco médicos o una tasa de 0,5 médicos por 1 000 habitantes antes del programa. Se procesaron los datos mediante la técnica de análisis de contenido*.

**Resultados.:**

*Se entrevistaron 30 licenciados y 28 técnicos en enfermería, 1 técnico administrativo y 19 agentes sanitarios. Los entrevistados valoraron positivamente el trabajo de los médicos cooperantes cubanos y resaltaron su responsabilidad, ética y humanismo, así como la elevada calidad de las consultas médicas y sus buenas relaciones con los pares de la atención básica*.

**Conclusiones.:**

*Se constataron diferencias en los patrones de atención de los médicos cooperantes cubanos del programa* Mais Médicos* con respecto a los médicos que ejercieron en las comunidades estudiadas antes de la implantación de ese programa. Entre los rasgos diferenciales de los médicos cubanos resaltados más frecuentemente figuran el compromiso con la población —tanto en la consulta médica como en la solución de sus problemas—, la empatía, el respeto y, en general, el humanismo con el que tratan a los pacientes*.

## Abstract (en)

**Objective.:**

To present the narratives of Brazilian members of family health teams with regard to the humanism perceived in the practice of Cuban physician cooperating in the *Mais Médicos* program.

**Methods.:**

Cross-sectional descriptive case study. A semi-structured interview was applied to Brazilian members of family health teams who had worked from the beginning of the program with Cuban physicians in selected municipalities included in the *Mais Médicos* program (20% or more of the population in extreme poverty and less than five physicians or a rate of 0.5 physicians per 1, 000 population prior to the program). The data were processed using the content analysis technique.

**Results.:**

Interviews were held with 30 senior nurses, 28 nursing assistants, one administrative technician, and 19 health agents. The interviewees offered a positive assessment of the work of the Cuban cooperating physicians, emphasizing their responsibility, ethics, and humanism, as well as the high quality of their medical -consultations and their good relations with their peers in basic health care teams.

**Conclusions.:**

There were confirmed differences between the patterns of care of the Cuban cooperating physicians in the *Mais Médicos* program and the physicians who were working before in the studied communities prior to implementation of that program. The differential features of the Cuban physicians most frequently mentioned include their commitment to the population (both in medical consultations and in solving people’s problems), their empathy, their respect, and in general, the human values with which they treat patients.

## RESUMO

**Objetivo.:**

Apresentar as narrativas dos integrantes brasileiros das equipes de saúde da família sobre o humanismo percebido na prática dos colaboradores cubanos do Programa *Mais Médicos*.

**Métodos.:**

Trata-se de um estudo de caso descritivo transversal. Foi realizada uma entrevista semiestruturada com os integrantes brasileiros das equipes de saúde da família que trabalhavam desde o início do programa com médicos cubanos em municípios selecionados cadastrados no Programa *Mais Médicos*. Estas localidades têm 20% ou mais da população vivendo em situação de extrema pobreza, contando com menos de cinco médicos (taxa de 0,5 médico por 1.000 habitantes) antes do programa. Os dados foram analisados com o uso da técnica de análise de conteúdo.

**Resultados.:**

Foram entrevistados 30 enfermeiros diplomados, 28 técnicos em enfermagem, 1 técnico administrativo e 19 agentes comunitários de saúde. Os participantes avaliaram de forma positiva o trabalho dos médicos colaboradores cubanos destacando características como responsabilidade, postura ética e humanismo bem como a alta qualidade das consultas médicas e o bom relacionamento com os pares da atenção básica.

**Conclusões.:**

Foram constatadas diferenças no padrão de atenção dos médicos colaboradores cubanos do Programa Mais Médicos em relação aos médicos brasileiros que atuavam nas comunidades estudadas antes da implantação do programa. Entre as características distintivas dos médicos cubanos destacadas estão o compromisso com os pacientes, tanto na consulta médica quanto na solução de problemas, a empatia, o respeito e o humanismo em geral com que eles tratam os pacientes.

En el Informe sobre Salud en el Mundo del año 2008 (1) se afirma que los sistemas de salud están creciendo en direc-ción contraria a la equidad y la justicia social, y se recomienda alinear las políticas en materia de recursos humanos y su formación con valores que promuevan esas aspiraciones en la práctica médica. La justicia social debería ser un contenido central de la formación ética en la carrera de medicina, ya que los médicos deben trabajar por el cambio en la sociedad para disminuir la inequidad. La protección de los derechos humanos mejora la calidad de los servicios de salud para todos (2).

La Organización Panamericana de la Salud (OPS) recomendó en el 2010 incluir entre las competencias básicas que deben cumplir los egresados de la carrera de medicina la capacidad de “establecer relaciones de respeto y confianza para dialogar y negociar con los ciudadanos/comunidad, equipos/comunidades y otros sectores, respetando la diversidad cultural” (3). Por lo tanto, la formación en valores humanistas debería ser uno de los ejes centrales a considerar en la formación de los médicos, que debe incluir el conocimiento del hombre pero, sobre todo, la capacidad de entender al hombre/prójimo/paciente con sus valores (4).

En salud pública, el humanismo se define como el conjunto de valores, actitudes y prácticas que consideran al paciente como un semejante que sufre y solicita alivio, y se basa, según algunos autores, en el afecto, el apoyo, el respeto y la solidaridad (5). Por su parte, la Junta Estadounidense de Medicina Interna (*American Board of Internal Medicine*) define como cualidades humanistas la preocupación profesional, la integridad, el respeto, la compasión, la responsabilidad profesional, la cortesía y la sensibilidad hacia el paciente para su bienestar y apoyo (6). Otros autores se refieren al humanismo como el respeto por la dignidad de las personas (7).

La considerable preocupación sobre la necesidad de incorporar valores de humanismo en los estudiantes de medicina ha quedado refrendada por numerosos autores (5–11). Esta preocupación parte de la marcada deficiencia en la aplicación de esos valores en la práctica profesional (5, 6). La lógica del mercado, asumida por una parte de la práctica médica en las últimas décadas, hace que se promuevan otras pautas —ligadas al lucro, la aplicación de tecnologías y la competencia (6-8)— que no siempre respetan los valores más elementales del humanismo. El modelo biomédico, que *“*cosifica*”* al paciente, atenta contra la comprensión humana del prójimo. La deshumanización de la atención médica es un reflejo de la deshumanización de la sociedad en general, donde el individualismo y la exacerbación del lucro están por encima de los valores humanos (5).

## Algunas características de la formación médica en Cuba y Brasil

En 1959, con el triunfo de la Revolución Socialista en Cuba, comienzan las transformaciones en el campo de la educación y la salud, y se adopta el proyecto de construir el derecho a la salud con una perspectiva universal y gratuita. En 1961, se creó el Ministerio de Salud Pública y se institucionalizó el nuevo sistema de salud cubano, acompañado de un proyecto educativo basado en nuevos valores sociales. En la década de 1980, se inició una reforma curricular dirigida a reorientar la formación médica hacia la atención primaria de salud (APS). Este proceso se vio favorecido por el hecho de que las universidades de enseñanza médica pasaron a depender administrativa y metodológicamente del Ministerio de Salud Pública (12).

Desde entonces, alrededor de 13% del plan curricular está enfocado a la APS (12) y en el currículo de medicina se incluyeron objetivos específicos y educativos. Entre estos últimos se encuentran los valores propios de la llamada educa-ción en el trabajo (12), que ocupa 60% de las 10 872 horas que componen el currículo de pregrado de la carrera de medicina en Cuba. En el marco de la educación en el trabajo, los estudiantes de medicina reciben como contenido transversal lo que denominan educación en valores —entre esos valores se incluyen la responsabilidad, la puntualidad, la comprensión y el respeto de los alumnos hacia los pa-cientes, apoyados en el ejemplo de los docentes—, así como contenidos específicos sobre ética médica (13).

En Brasil, la formación médica se inició en la época en que la monarquía portuguesa se trasladó a Río de Janeiro, en 1808, cuando se abrieron los cursos de cirugía y anatomía. Según Neves, en 1812 se realizó la primera reforma de la formación médica; no obstante, en ella no se incluyó la enseñanza de la ética, aun cuando se incorporó la medicina legal (14). No fue hasta octubre de 1969, con la aprobación de la Resolución 8 (15), que se introdujo la ética médica como disciplina en el currículo de la carrera de medicina. No obstante, la formación en esta materia continuó siendo teórica, poco humanista y basada en la especialidad, con un perfil individualista centrada en el hospital (14). Recién en el año 2013, a partir de la creación del programa *Mais Médicos*, se estableció la obligatoriedad de que los cursos de medicina se guiaran por las Directrices Curriculares Nacionales de la Carrera de Medicina (Ley 12.871, artículo 4) (16), con una declaración explícita de basar la formación médica en principios éticos y humanistas.

Según Muñoz y Muñoz, antes de los más recientes cambios curriculares del año 2014 en Brasil, el prestigio social de los médicos no había aumentado en la misma proporción que los avances en las ciencias médicas. Estos autores describieron los frecuentes reclamos presentados ante los consejos regionales de medicina, en el sentido de que las prácticas de estos profesionales eran poco humanas y carentes de una conducta digna (17).

## El programa *Mais Médicos*

El Gobierno de Brasil creó el programa *Mais Médicos* el 8 de julio de 2013 y este quedó establecido mediante la Ley 12.871 de octubre de ese año (16). Su objetivo era ampliar la accesibilidad y la cobertura del Sistema Único de Salud (SUS) en las zonas del país con mayor carencia de profesionales y formar un contingente amplio de médicos mediante la apertura de nuevas plazas en las facultades de medicina.

Para que los médicos pudieran participar en el programa, la Ley 12.871 estableció criterios de prioridad que, aunque no eran excluyentes, debían ser atendidos. El Gobierno determinó que se daría preferencia a los médicos brasileños formados en Brasil y en el extranjero, y después a los médicos foráneos. Fruto de la cooperación internacional bajo la gerencia de la OPS, al programa se incorporaron 11 439 profesionales cubanos de la salud, que pasaron a formar parte de los equipos de la Estrategia de Salud de la Familia, que es la forma en que se organizó la APS en Brasil. Estos equipos están compuestos por un médico, un enfermero licenciado, un técnico de enfermería y agentes comunitarios, aunque algunos pueden tener también un dentista y un asistente dental. Cada equipo está adscrito a un centro de salud y atiende a la población que vive en el territorio específico asignado.

El presente estudio se enmarca en un proyecto más amplio denominado “Análisis de la efectividad de la iniciativa Mais Médicos en la realización del derecho universal a la salud y la consolidación de las redes de servicios de salud”, auspiciado por el Consejo Nacional de Desarrollo Científico y Tecnológico (CNPq) y por el Departamento de Ciencia y Tecnología, del Ministerio de Salud de Brasil (DECIT/MS). El objetivo de este artículo es mostrar de manera sistematizada las narrativas de profesionales brasileños que trabajan en los equipos de salud de la familia en Brasil acerca del humanis-mo percibido en la práctica de la aten-ción primaria de los médicos cubanos del programa Mais Médicos.

## MATERIALES Y MÉTODOS

Se trata de un estudio de caso descriptivo de corte transversal, basado en una metodología cualitativa (18). El trabajo de campo se realizó durante el año 2015.

La muestra para entrevistar se seleccio-nó en varias etapas. En primer lugar, se definió el número posible de municipios a visitar, del total de 3 785 municipios brasileños donde funcionaba el programa *Mais Médicos* en 2014, se escogieron 32; los criterios de inclusión de los municipios fueron: tener 20% o más de la población en extrema pobreza, estar inscrito en el primero o el segundo ciclo de la convocatoria del programa, y tener menos de cinco médicos o una tasa de 0,5 médicos por 1 000 habitantes antes de la implementación del programa en la zona. Se aplicó un muestreo aleatorio simple de los municipios que cumplían estos criterios en cada una de las regiones de Brasil. Una vez escogidos los municipios, se realizó un muestreo intencional para elegir los equipos que serían entrevistados, a partir de su disponibili-dad. De cada equipo de salud de la familia seleccionado se entrevistaron al menos dos miembros de nacionalidad brasileña que trabajaran desde el inicio del programa con médicos de nacionalidad cubana.

Las entrevistas, basadas en un cuestionario semiestructurado, se grabaron y transcribieron. Las entrevistas cubrían siete grandes temas: la relación con el médico y el trabajo en equipo, la percepción de cambios en la consulta médica después de la implantación del programa, la percepción de cambios en la red de atención, los servicios diagnósticos, el equipos de apoyo profesional, las contribuciones que ellos creían que el programa *Mais Médicos* estaba dando a la comunidad y, por último, se pedía una evaluación sobre lo que podría mejorarse en el programa. Para el procesamiento de los datos se utilizó la técnica de análisis de contenido (19) mediante el ordenamiento de los datos en categorías y códigos, delimitados según la emergencia de los temas; el análisis se realizó por códigos. Dos investigadores codificaron los datos de manera independiente y llegaron a un criterio similar. Las pequeñas discrepancias que surgieron en la codificación del material se dirimieron en una reunión del equipo de investigación.

Aunque las preguntas no estaban dirigidas explícitamente al humanismo del modelo de atención de los médicos cubanos y su comparación con el brasileño, en todas las entrevistas surgieron numerosas narrativas que apuntaban a esto, por lo que se utilizó como foco para el análisis.

Esta investigación recibió la aprobación del Comité de Ética de la Facultad de Ciencias de la Salud de la Universidad de Brasilia (No. 399.461). Cada participante firmó una declaración de consentimiento libre e informado. Se to-maron medidas para garantizar la confi-dencialidad de los participantes.

## RESULTADOS Y DISCUSIÓN

Se seleccionaron municipios de las cinco regiones de Brasil: 14 del nordeste, 12 del norte, 3 del sudeste, 2 del centro-oeste y 1 del sur. Se realizaron 78 entrevistas en total. Se entrevistaron 30 licenciados y 28 técnicos de enfermería, 1 técnico ad-ministrativo y 19 agentes sanitarios. La gran mayoría (80,8%) de los entrevistados eran mujeres.

Las narrativas de los miembros brasi-leños de los equipos de salud de la familia sobre el trabajo de los médicos cooperantes cubanos tienen un sentido positivo, contrariamente a las narrativas recogidas sobre los médicos brasileños actuantes en la región antes del programa. Estas diferencias se manifestaron en los relatos mediante frases como *“los brasileños”, “los cubanos”,* *“el médico anterior”* o *“cuando teníamos médico”*. Se concluyó que con esas frases se identificaban dos modelos de atención con valores humanistas diferentes. La desconstrucción de este discurso, sin generar estigmas, fue la base para la reflexión sobre los valores de humanismo que los miembros de los equipos de salud de la familia imaginan con carácter positivo para los usuarios y para el trabajo en conjunto.

Se presentan a continuación algunos tópicos expresados por los entrevistados, que se refieren a esas diferencias.

Uno de los problemas enfrentados por las poblaciones que participan del programa *Mais Médicos* era la dificultad de contar con atención médica de forma sistemática y continua. El personal entrevistado se refirió al escaso tiempo que los médicos anteriores a la llegada de los médicos cooperantes pasaban en el municipio, y relacionaron este hecho con la falta de vínculo con la comunidad:

*“No conseguían tener vínculo con ese médi-co, dentro de los tres meses salían y dejaban una laguna”* (E45).

El tema de la alta rotación de médicos dentro de la Estrategia de Salud de la Familia está documentado por diversos autores (20-22). Como causas de este problema se señalan la baja satisfacción de los médicos con ese trabajo, los problemas derivados de la gestión política y partidaria de los municipios, y la precariedad en las formas de contratación. La actuación como médicos de familia se ve como un lugar vacío de prestigio o temporal (23).

A partir de la llegada del programa *Mais Médicos,* la permanencia de los médicos mejoró:

*“Este proyecto está siendo muy bueno porque antes estábamos años sin médico”* (E73).

Según los relatos, los médicos cubanos, además de dar una continuidad al trabajo, contemplaban la dimensión hu-mana, en este caso, la comprensión del sufrimiento humano tras una necesidad de atención:

*“El médico […] no manda irse a las personas [sin ser atendidas], atiende a todo el mundo, la ciudad solo gana…”* (E7).

*“Esa preocupación con el paciente, fue una mejoría del 100%”* (E23).

*”El compromiso que tiene es bien diferente. Nunca vi un profesional tan preocupado”* (E52).

En estas narrativas, son los médicos anteriores los que dejaban gente sin atender y los que no expresaban su preocupación. En otros relatos, esto se expresa directamente:

*“Después de que la doctora llegó, no vimos más gente sin atender en esta unidad”* (E61).

*“Mejoró bastante porque la preocupación del cubano es bien diferente de la de un brasile-ño”* (E76).

La preocupación por el paciente, forma parte de la empatía que significa saber apreciar los sentimientos del otro (24) y esta constituye una característica propia del humanismo (5).

Un importante factor de apreciación positiva se revela en el tiempo destinado a la consulta:

*“Mejoró mucho porque antes, aquella cosa, aquella corrida, quiere estar en tres lugares al mismo tiempo […] Desde que el médico cubano está aquí todo el día, la calidad del servicio mejoró bastante”* (E4).

Los actores entrevistados se referían con “corrida” a la falta de cumplimiento del horario de atención pese a su gran demanda, aun cuando la contratación para la atención primaria es de dedicación exclusiva.

Según un estudio sobre la APS en Brasil, los médicos de la Estrategia de Salud de la Familia, aun cuando tenían sus 40 horas cubiertas, poseían otros trabajos (22). Otra investigación, realizada en un municipio del nordeste brasileño, concluyó que los profesionales de la atención primaria procuraban otros vínculos laborales como forma de complementación salarial, a pesar de tener contratos de dedicación exclusiva (25). La acumulación de lucro a costa de la calidad del trabajo profesional va en detrimento del compromiso con las personas, las familias y las comunidades. La ética humanista aboga por una medicina centrada en el paciente y no en el lucro económico (24). Por ello, la práctica médica en la APS debería incorporar entre sus conte-nidos humanistas la formación en derechos humanos y la justicia social para revertir las inequidades en la salud —producto de la inequitativa distribución de la renta— y no reproducirlas (2).

Al hablar sobre los médicos cooperantes cubanos, los entrevistados remarcaron que, además de dedicar mayor tiempo, se preocupaban por la solución del problema:

*“La persona consulta y ellos ya marcan un retorno… entonces la gente ya siente mejoría por eso (…), mejoró bastante debido a tener esa atención, esa preocupación”* (E23).

La diferencia entre los médicos cubanos cooperantes y lo que los profesionales y técnicos relatan sobre los “anteriores” podría explicarse por esa preocupación, que en la ética humanista se relaciona con la empatía. En un estudio longitudinal realizado en Filadelfia, Estados Unidos de América, durante seis años, se evidenció que en el tercer año de la formación médica hay un descenso de la empatía en los estudiantes de medicina. El argumento que expresan los alumnos es que lo ven en sus superiores y eso afecta a sus propias experiencias (26). En este sentido, cabe recalcar que, antes de la reforma, la cantidad de horas dedicadas a la ética en el currículo médico brasileño dependía del centro formador. En un estudio realizado en dos reconocidas universidades brasileñas, estos contenidos consagraban a ética médica entre 2,1% y 2,7% del total de horas (27), mientras que en el currículo de formación de médicos en Cuba ocupa 36 horas (0,33%) como contenido específico, pero es un contenido transversal en la educación en el trabajo, cuya carga horaria ocupa, como ya se ha dicho, 60% de la formación total (28) e incluye explícitamente el ejemplo del profesor como parte fundamental del aprendizaje humanista (29).

Cuando los técnicos y profesionales hablan positivamente de la capacidad de resolución de los médicos del programa y la contraponen a la de los “médicos anterio-res”, se refieren a los siguientes aspectos:

- el retorno a la consulta médica
  *“… mientras que la presión no quede normal, el médico no deja de atender, y el brasileño no hace eso”* (E41).
- la contrarreferencia
  *“... conseguimos derivar y tener respuesta, porque antiguamente solamente derivábamos pero no teníamos respuesta”* (E5).
- la observación de los pacientes
  *“Llegó un bebecito de cuatro meses y una madre que no vivía en el municipio y ella [la doctora] se sentó para conversar y miró al bebé y no direccionaba la mirada y cuando vimos, el bebé era ciego… ya había pasado por tres centros de salud y nadie percibió que el bebé no miraba”* (E11).

La comparación realizada por los -entrevistados destaca el compromiso y la empatía de los médicos cubanos. Si bien estas narrativas forman parte del deber profesional de un médico, estaban ausentes en experiencias anteriores, según el relato de los profesionales entrevistados.

Los actores entrevistados observaron numerosos elementos para analizar la satisfacción actual con los médicos cooperantes del programa, comparada con la que tenían anteriormente. La mayoría de los entrevistados destacó aspectos del cuidado dentro de la consulta, tales como:

- escuchar
  *“Ella escucha, ella realmente atiende como tiene que ser, humanizado” (E6)*.
- mirar a la cara
  *“Los médicos de aquí, brasileños, ni mirar a la cara del paciente; él si mira” (E78)*.
- conversar
  *“Cuando era el otro médico, no tenía tiempo de conversar con el paciente… ella no, ahora quiere saber del padre, la madre y toda la historia del paciente” (E4)*.
- examinar
  *“Él examina directo a todos los pacientes, lo que es una realidad bien diferente de los médicos brasileños” (E7)*.

En un estudio realizado en un centro de salud de la ciudad de Palmas, en el estado Tocantins, Brasil, se encontraron resultados similares a los hallados en este estudio (30). La insatisfacción con la atención recibida era debida a la falta de tiempo para una consulta adecuada, el incumplimiento de la carga horaria de los médicos, no escuchar al paciente adecuadamente, no hacer exámenes físicos, y dar la sensación de indiferencia y de poco vínculo con los problemas que se les presentaban (30). Los elementos subjetivos parecen formar parte de la evaluación, ya que expresan aspectos clave de la atención médica basada en preceptos humanistas (bajo la forma de preocupación por resolver el sufri-miento de otro, dignidad y empatía por el otro). El éxito de una buena relación médico-paciente radica, entre otros elementos, en la preocupación que el médico manifieste al enfrentarse con el sufrimiento del paciente y en la capacidad que demuestre para satisfacer las expectativas de alivio y cuidado del enfermo (31). El aporte de una ética humanista en el trabajo de los profesionales médicos redunda en una mejor calidad asistencial.

Los valores humanistas implican el res-peto por la dignidad humana, la preocupación por el otro, la empatía y el reconocimiento del semejante que sufre y solicita alivio. La incorporación de valores humanistas a la práctica médica contribuye a la justicia social y la equidad en la salud, ya que se reconoce al otro como semejante, con sus especificidades y en su plena di-mensión de portadores de derechos.

Los médicos de Cuba tuvieron desde la década de 1980 una formación desde y para la atención primaria, con contenidos transversales en valores humanistas que ocuparon más de la mitad de sus estudios. Por su parte, la formación de los médicos brasileños tuvo un perfil biomédico bas-do en el paradigma flexneriano y no fue hasta el año 2014, después de la promulga-ción de la ley que institucionalizó el pro-grama Mais Médicos, que se establecieron directrices para reforzar el perfil de la ca-rrera de pregrado hacia la formación de médicos generales y se incorporó la obligatoriedad de un internado en APS. Estas diferencias en la formación, sumadas a las diferencias de los sistemas sociales y económicos de Brasil y Cuba, podrían ex-plicar los contrastes hallados entre los mo-delos de atención, reflejados en las narrativas de los técnicos y profesionales entrevistados. Se debe reiterar que si bien en general las narrativas se reducen a la na-cionalidad, de lo que en realidad se trata es de la caracterización de dos modelos de atención. A los entrevistados no les pasa-ron inadvertidas las diferencias en las ca-racterísticas del trabajo de los médicos cooperantes cubanos ni lo que ellos creen que es un trato digno para la población que utiliza los servicios de atención básica de salud.

La formación de médicos para la APS con valores humanistas constituye una preocupación en el nuevo milenio.

Al analizar estos resultados se deben tener en cuenta algunas limitaciones. Por tra-tarse de un estudio descriptivo, no se sugieren esquemas de causalidad en la in-terpretación de datos; simplemente se trata de un análisis de datos en un contexto de prácticas y formación. Otra limitación es el posible sesgo de memoria a la hora de referirse a los *“*médicos anteriores*”*. Por último, los relatos de los encuestados aluden a los médicos brasileños que trabajaban en la zona antes de la implementación del programa *Mais Médicos*, lo que puede haber variado en la actualidad.

## CONCLUSIONES Y RECOMENDACIONES

Se constataron diferencias en los patro-nes de atención de los médicos cooperantes cubanos del programa *Mais Médicos* con respecto a los médicos que ejercieron en las comunidades estudiadas antes de la implantación de ese programa. Entre los rasgos diferenciales de los médicos cubanos resaltados con mayor frecuencia por los entrevistados figuran el compromiso con la población —tanto en la con-sulta médica como en la solución de sus problemas—, la empatía, el respeto y, en general, el humanismo con el que tratan a los pacientes.

Reflexionar sobre el modelo de profesionales que necesita un sistema de salud, en este caso el SUS, desde la óptica de quienes cotidianamente trabajan en la atención primaria, contribuye a profundizar en los saberes y las prácticas para producir un encuentro mejor logrado entre los servicios de salud y la población. Esta reflexión también contribuye a abrir nuevas perspectivas de investigación en un tiempo en el que es preciso colocar el humanismo en las agendas políticas y de salud, especialmente cuando se trata de poblaciones en situación de vulnerabilidad, frecuentes en las socie-dades contemporáneas.

### Agradecimientos.

Esta investigación recibió financiamiento de la Convocato-ria Pública MCTI/CNPq/CT-Saúde/MS/SCTIE/Decit Nº 41/2013 del CNPq y DE-CIT, de Brasil. Los patrocinadores no participaron de ninguna manera en el diseño del estudio, la colecta y el análisis de los datos, la decisión de publicar este trabajo, ni la preparación del manuscrito.

### Declaración.

Las opiniones expresadas en este manuscrito son responsabilidad de los autores y no reflejan necesariamente los criterios ni la política de la *Revista Panamericana de Salud Pública/Pan American Journal of Public Health* o de la Organi-zación Panamericana de la Salud.
